# Autoimmunity After Ischemic Stroke and Brain Injury

**DOI:** 10.3389/fimmu.2019.00686

**Published:** 2019-04-02

**Authors:** Ehsan Javidi, Tim Magnus

**Affiliations:** Department of Neurology, University Medical Center Hamburg-Eppendorf, Hamburg, Germany

**Keywords:** autoimmunity, traumatic brain injuries (TBI), autoantibodies, ischemic stroke (IS), adaptive immunity, neural antigens, autoreactive T and B cells, neuroinflammation

## Abstract

Ischemic Stroke is a major cause of morbidity and mortality worldwide. Sterile inflammation occurs after both stroke subtypes and contributes to neuronal injury and damage to the blood-brain barrier with release of brain antigens and a potential induction of autoimmune responses that escape central and peripheral tolerance mechanisms. In stroke patients, the detection of T cells and antibodies specific to neuronal antigens suggests a role of humoral adaptive immunity. In experimental models stroke leads to a significant increase of autoreactive T and B cells to CNS antigens. Lesion volume and functional outcome in stroke patients and murine stroke models are connected to antigen-specific responses to brain proteins. In patients with traumatic brain injury (TBI) a range of antibodies against brain proteins can be detected in serum samples. In this review, we will summarize the role of autoimmunity in post-lesional conditions and discuss the role of B and T cells and their potential neuroprotective or detrimental effects.

## Introduction

Ischemic Stroke and traumatic brain injury (TBI) cause damage to neurons, glia and the vasculature resulting in the disruption of the blood-brain barrier (BBB), hemorrhage, edema, and necrotic cell death. Cell injury and cell death lead to the release of danger signals that activate the immune system (1, 2). The subsequent sterile inflammatory reaction involves the innate immune system with activation of resident immune cells of the central nervous system (CNS) and a rapid infiltration of peripheral immune cells into the brain (3, 4). The presence of brain-derived antigens in the lymphoid tissue of stroke patients (5) and increased levels of brain antigens in the CNS and peripheral circulation in both diseases may trigger an adaptive autoimmune response. This process would require the presentation of antigen by antigen-presenting cells (APCs) to autoreactive T cells. The prevention of detrimental autoimmunity is usually mediated by central and peripheral tolerance mechanisms which include anergy, clonal deletion and suppression by regulatory T cells (Tregs) (6). Self-reactive T and/or B lymphocytes and/or their ability of autoantibody production can exist as physiological autoimmunity with no evidence of clinical disease, demonstrated by the presence of natural autoantibodies that are involved in homeostasis by degrading self- and foreign antigens (7). In the inflammatory microenvironment following stroke and TBI, self-reactive lymphocytes and autoantibodies could be generated and participate in inflammation, when immune tolerance is broken, leading to pathological autoimmunity, and further tissue injury. This antigen-dependent adaptive autoimmune response would therefore differ from the reactive innate immune response that involves antigen-independent deleterious effects of T cells found in acute experimental stroke (8). The presence of brain-reactive antibodies in stroke and TBI could be therefore an indication of an autoimmune response and multiple investigations now demonstrate their correlation to lesion size and functional outcome in stroke and TBI.

## Antigen Specificity in Ischemic Stroke

Ischemic stroke leads to a sterile inflammatory response with the accumulation of microglia, and infiltration of macrophages, lymphocytes, and dendritic cells (DCs) followed by neutrophils (3). T lymphocytes can be found in human post-mortem stroke tissue to a small extent in necrotic brain parenchyma and more consistently in perivascular spaces bordering acute infarcts (9). The activation of T lymphocytes via an antigen dependent or independent process is still controversial and the mechanisms of T cell activation after brain injury are not clearly known (10).

Stroke causes the disruption of the blood-brain barrier (BBB): Infiltrating neutrophils, major promoters of BBB breakdown, through the release of metalloproteinases (MMP) such as MMP-9, together with inflammatory mediators, lead to the degradation of tight junction (TJ) proteins (11, 12). This first pathway could allow for the release of brain specific antigens into the peripheral vasculature, depending on the degree of BBB permeability and the size of brain antigens. A second pathway through the glymphatic system could allow for cerebrospinal fluid (CSF) drainage of interstitial fluid (ISF), containing extracellular solutes such as brain antigens, from the brain parenchyma, along paravenous pathways into the lymphatic circulation, eventually reaching the lymph nodes (13, 14). The brain lymphatic vessels lining the dural sinuses, drain to the deep cervical lymph nodes with the capability of carrying fluids, immune cells, and possibly brain antigens from the CSF (15, 16).

The accumulation of APCs and the upregulation of MHC II molecules in the ischemic brain (1, 17, 18) coinciding with the peak infiltration of lymphocytes into the brain (3), could allow for an antigen-dependent T cell expansion in the brain itself. In experimental ischemic stroke, clonal T cell expansion could be detected after 7 and 14 days following stroke (19). APCs with brain antigens could also travel through the glymphatic route and brain meningeal lymphatic vessels into the lymph nodes. Providing that significantly more brain-derived antigens can be found in cervical lymph nodes of stroke patients (5), monkey experimental autoimmune encephalomyelitis (EAE) and individuals with multiple sclerosis (MS) (20) than in controls, the possibility of T cell reactivity to autoantigens through activation by antigen-presenting cells (APC) in the periphery and subsequent migration into the brain also exists. T cells monitoring the leptomeningeal surfaces could also be activated by CNS antigens presented by phagocytes, or re-stimulated by CNS antigens after being primed by antigens in the periphery (21, 22).

The development of an immune response or tolerance to brain antigens presented by APCs in lymph nodes would depend on several factors such as the activation status of APCs, costimulatory molecules, the environment of antigen presentation, or even a genetic predisposition to autoimmune disease (23, 24).

The role of dendritic cells (DCs), as professional antigen presenting cells, therefore deserves closer attention. DCs play a central role in maintaining immune tolerance. In a non-inflammatory environment, immature DCs present self-antigens to T cells, leading to anergy or deletion of T cells to avoid autoimmunity. Immature DCs have also been found in the meninges and the choroid plexus of the CNS sampling the CSF for antigens (25). The activation of DCs by pathogen or danger-associated molecular patterns (DAMPs), leads to DC maturation and migration into draining lymph or the ischemic brain, with an increase of MHC-II and co-stimulatory proteins, relevant for T cell activation (26, 27). In experimental autoimmune encephalomyelitis (EAE), different subtypes of activated DCs with upregulated levels of costimulatory molecules are abundantly present in inflamed CNS lesions and CSF, driving proinflammatory T cell responses (28, 29). The model of EAE is induced by the priming and expansion of CD4+ T cells responding to CNS myelin antigens and the disease is initiated by re-presentation of myelin epitopes by APCs (30). Peripherally derived myeloid dendritic cells (mDCs) in EAE were found to present endogenous myelin peptide, activating naive autoreactive pathogenic CD4+ T cells to proliferate and produce IL-17 (30). Recently, we showed that the ischemic brain is rapidly infiltrated by IRF4^+^/CD172a^+^ conventional type 2, IL-17 inducing DCs. These cells promote neutrophil infiltrating into ischemic hemispheres via induction of IL-17 in γδ T cells, which can be activated in a T cell receptor-independent manner (27). The question whether this DC population or another subtype present in the stroke brain, could mitigate an autoimmune response to brain antigens comparable to EAE, escaping tolerance mechanisms or becoming activated after re-encountering naturally occurring antibodies, therefore warrants attention in further studies.

Microglia, as the major resident immune cells in the brain become activated through proinflammatory signaling as a result of brain ischemia (31). Activated microglia are involved in cytokine production, phagocytosis, and antigen presentation (1). In multiple sclerosis (MS) patients and its animal models (EAE), activated microglia and infiltrating T cells are found in CNS lesion (32). Activated microglia act as APCs by expressing MHC class I and II together with costimulatory molecules (33). Their role as antigen-presenting cells in stroke has received only little attention until recently. In a study of experimental brain ischemia, microglia-like cells acted as the main antigen-presenting cells for myelin oligodendrocyte glycoprotein (MOG) antigens, leading to the expansion of previously transferred 2D2 reactive CD4+ T cells. Higher frequencies of MOG-specific T cells were found in the brain at day 4 and 7 after middle cerebral artery occlusion (MCAO) (34). MHC class II/TCR interaction was necessary for T cell expansion in the brain with the highest expression of MHC class II found on microglia-like cells. The T cell expansion was dependent on cerebral ischemia. This antigen-dependent T cell response resulted in increased neurodeficits and infarction volumes starting at day 4 after MCAO (34).

The time course of immune cell infiltration after stroke needs to be considered in distinguishing between possible antigen-dependent autoimmune and antigen-independent responses. Lymphocyte infiltration after experimental stroke has been suggested to take place as early as 3 h for CD8+ T cells and 24 h for CD4+ T cells (35–37), while other publications report a delayed peak of CD4+ T cells at 3 days (3, 38) or 5 days (39). B and T lymphocytes are present in different models of stroke up to 7 weeks after stroke onset (40).

T cells in the acute phase of experimental stroke have been linked to lesion volume and functional outcome (1, 41). Transgenic mice deficient of lymphocytes and antibody-mediated depletion of CD4+, CD8+, and γt T cells have been associated with smaller infarcts and better functional outcome (8, 39, 42–45).

A possible antigen-specific T cell response to brain antigens however would take place several days after stroke according to the time that antigen uptake, processing, presentation and finally lymphocyte proliferation would need. The attenuation of secondary stroke injury after experimental stroke by interventions targeted at T cells has been repeatedly shown to be within the first days. Early detrimental lymphocyte invasion has been therefore deemed antigen-independent within the first days after stroke onset (8).

Fittingly, a study of tMCAo stroke in mice, showed a significant autoimmune response to brain antigens at 4 days through 10 days after stroke, determined by an increase in autoreactive CD4+ and CD8+ T cells and CD19+ B cells, isolated from spleens and cervical lymph nodes (46). The peak of autoreactivity was reported to be at 8 days in splenic CD4+ T cells and at day 10 for CD8+ T cells in the cervical lymph nodes. An increased autoreactive response to microtubule-associated protein 2 (MAP2) and myelin derived peptides such as myelin basic protein (MBP) and MOG was associated with smaller infarct volumes and an increased autoreactive response to the anti N-methyl-D-aspartate receptor 2A (anti-NMDAR2A) with larger infarct volumes in mice (46).

In contrast to (46), a study of brain-derived antigens in the cervical lymph nodes and palatine tonsil of 22 patients with acute stroke, found increased immunoreactivity to neuronal MAP2 and NMDA NR-2A to be associated with smaller infarct volumes at day 7 and better outcome, whereas increased immunoreactivity to myelin-derived MBP was associated with more severe impairments on admission, larger infarcts and worse outcome at clinical follow-up (5). Cells immunoreactive to neural antigens were mostly CD68+ macrophages expressing costimulatory MHC II receptors and their co-localization with CD69+ T cells suggested activation of T cells (5).

There is further evidence for detrimental antigen-dependent effects: CD8+ T cells with a ovalbumin-specific T cell receptor were transferred into Rag1 mice showing no activation, less brain infiltration and smaller infarct sizes at 7 days after pMCAO than after the adoptive transfer of wild-type CD8+ T cells which could function antigen-dependently (45).

Another important question is the role of regulatory T cells (Tregs). The role of Tregs in the regulation of lymphocytic activity especially in light of exposure to neural antigens has not been fully answered. Tregs have a role in controlling autoreactive responses and have been shown to inhibit autoimmunity, maintaining peripheral tolerance (47). Following stroke, an early infiltration of Tregs (CD25+Foxp3+) was observed within the first week after MCAO in mice, constituting 20% of all CD4+ T cells by Liesz et al. (48), while we found only <5% of all CD4+ T cells to be Tregs within the first week (3). Accordingly, (49) found a substantial infiltration of Tregs to be at 14 and 30 days as compared to 7 days after MCAO. Tregs, despite their delayed infiltration, have been nevertheless found to impact stroke outcomes within the first days after ischemia. It has been suggested that Tregs could target cells of the peripheral immune system early after ischemia, suppressing peripheral T cell activation or inhibiting autoantigen-specific clonal expansion (50). Accordingly, an increase of Tregs can be seen in the spleen following MCAO with possible implications on post-stroke immunosuppression (51). The question whether Tregs in stroke are neuroprotective or detrimental also remains disputed (52). A recent review found the majority of studies with Tregs targeted therapies to support the beneficial role of Tregs, with reduction of lesion size. Treg depletion models were more likely to show an increased infarct volume and increased leukocyte brain invasion and proinflammatory cytokine secretion, however some studies showed no effect on infarct volume (50). The difference in outcome has been attributed to the different intensity of neuroinflammation dependent on the lesion sizes in the use of different ischemia models (50). In a recent study, a substantial accumulation of Tregs in the mouse brain was detected starting a week after ischemic stroke, potentiating neurological recovery. Tregs transferred from ischemic mice accumulated more efficiently in the brain of lymphocyte deficient Rag2 ^−/−^ mice than Tregs from sham operated mice, suggesting the importance of antigen recognition for Treg cell expansion in the brain (53). The involvement of Tregs in suppressing autoimmune responses to neural antigens can be deduced from studies that use tolerization to brain antigens, showing an increased probability of Treg responses and improved neurological outcome (54–56). These studies are discussed further below.

Taken together, there evidence of an antigen-specific T cell presence in ischemic stroke. This develops within several days and decreases over time. It is unclear however if these findings are relevant to the human stroke.

## T Cells: Antigens and Tolerance in Stroke

There is evidence for T cell reactivity to autoantigens such as myelin basic protein (MBP) and myelin proteolipid protein (PLP) in patients with ischemic stroke (57). Higher peak concentrations of MBP, neuron-specific enolase (NSE) and S100B in the serum of patients at 24 h after stroke have been associated with stroke severity and larger infarct volumes (58).

If T cells, particularly CD4+ T cells, encounter an antigen, they can react in several ways. Depending on the cytokine milieu the effector function of T cells will be shaped (59). For example, a TH1 type response, a TH2/TH3 response, a TH17 response, an activation-induced cell death or no immune response can follow. A TH1 response (sensitization) leads to the secretion of proinflammatory cytokines after reencountering with the sensitizing antigen. A TH2/TH3 response (tolerance) leads to the secretion of immunomodulatory cytokines after reencountering with the antigen. Stroke induces an early systemic inflammatory response, which could promote sensitization to brain antigens in an altered microenvironment (60). It is believed that mucosal antigen administration shifts the immune response from a TH1 response with secretion of proinflammatory cytokines (e.g., IL-1b, TNF-α, IFN-y) to a TH2 or TH3 response, with secretion of anti-inflammatory cytokines (e.g., TGF-b1, IL-10) that would limit cell-mediated responses and enhance humoral responses. In addition, antigen-specific regulatory T cells can produce anti-inflammatory cytokines after encountering a previously administered autoantigen (60, 61). There is evidence that TH1 type responses to MBP are associated with worse neurological outcome following experimental stroke (60, 62, 63). In one study, the adoptive transfer of MBP specific TH1 (+) or TH17 (+) cells into naïve recipient mice resulted in a worse neurological outcome (63). The adoptive transfer of splenocytes reactive for MOG into immunodeficient MCAO mice led to their migration into the ischemic hemispheres and resulted in an increased infarct volume (64). However, TH1 responses to antigen stimulation with MBP following 1 month after experimental MCAO were low and animals were more likely to develop a TH2/TH3 type response (60). Treatment with the pathogenic lipopolysaccharide (LPS) to stimulate a systemic inflammatory response, showed an increased sensitization to MBP with an increased TH1-response and more severe neurologic deficits and brain atrophy at 1 month after MCAO. This suggested that a systemic inflammation after stroke could lead to a detrimental autoimmune response (60, 65). LPS also induced the expression of MHC II molecules on microglia required for lymphocyte activation which are usually not expressed in the CNS (66). In a more recent study of the same group, which investigated autoimmune responses to MBP after stroke following immunization with MBP, again only 1/4–1/3 of the animals had a TH1 or TH17 response to MBP. Those with a response were also associated with worse outcome at 1 month (67). The detrimental effect was observed shortly after experimental ischemia. Animals that were sensitized to MBP prior to stroke had a sharply increased mortality within 24 h, whereas induction of oral tolerance to MBP resulted in significantly reduced infarct sizes at 24 h and 96 h after ischemia (68). MCAO animals who received splenocytes from MBP tolerized donors showed a decrease in infarct volume (69). Animals who were tolerized to MBP before MCAO induction and LPS treatment were less likely to develop a TH1 response to MBP than ovalbumin (OVA) tolerized animals and had an increased probability of a TH3 or TREG response together with smaller infarct sizes after 1 months (55). Similarly, mice who were previously nasally tolerized to MOG showed a significant shift from the proinflammatory cytokine IFN-y to the anti-inflammatory cytokine IL-10 after MCAO induction (61). Adoptive transfer of CD4+ T cells from nasally tolerized mice to wild-type mice showed a significant reduction in infarct size. This effect could not be observed in IL-10 deficient mice (61). Treatment with a neuroantigen-specific peptide such as recombinant T cell receptor ligands (RTL), linked to myelin peptide (MOG), reduced lesion size and inhibited the accumulation of inflammatory cells, presumably by inducing tolerance to MOG (70). RTL specifically targeting myelin-specific T cells can modulate their characteristics to anti-inflammatory cells that secrete IL-10 (71).

Similar observations have also been made in clinical studies. In a study with 114 patients of ischemic stroke, a strong TH1 response to MBP at 90 days was associated with worse outcome and was more likely to develop in patients with severe stroke and post-stroke infection (72). The degree of the TH1 response however decreased over time, pointing to the possibility of immunoregulatory mechanisms (73). Recently, in brain slices from 5 patients with acute middle cerebral artery ischemic stroke, who died within 7–10 days after stroke onset, T cells, including MOG-specific CD4+ T cells were found in the infarct and peri-infarct area close to ischemic neurons (34). In a cohort of 28 patients with milder ischemic stroke, frequencies of MOG and MBP-specific IFN-γ responses, that were measured in peripheral blood, were increased early within the first week after stroke and decreased at 3 months. Naïve CD4+ and CD8+ T cells were also decreased within the first days after stroke, which was attributed in the context of stroke-induced immunosuppression (SIDS) to a possible adaptive mechanism, preventing long-term autoimmune responses (74). Accordingly, blockade of SIDS in transgenic mice with the CD4+ MOG T cell receptor, resulted in increased antigen specific TH1 responses at 14 days after MCAO (75).

Repetitive mucosal tolerance with low doses of antigen is known to generate regulatory T cells and suppress EAE by inducing peripheral tolerance (76). In one study, repetitive mucosal tolerance to E-selectin, a glycoprotein adhesion molecule expressed on activated endothelial cells after stroke, suppressed TH1-mediated delayed-type hypersensitivity (DTH) reaction to E-selectin, and increased IL-10 secretion in adoptively transferred splenocytes from tolerized animals. Reduced infarct sizes could be detected as early as 6 and 48 h after MCAO (54). In another study of mucosal tolerance to E-selectin, an increased neurogenesis together with increased Tregs in the cervical lymph nodes and peri-infarct region were found after pMCAO together with an improved behavioral recovery at 2–4 weeks (56).

Taken together, antigen specific tolerance can be used in some animal models to lessen stroke damage. These experiments show tolerization, however the exact mechanisms are not fully understood. This could be T cell modifying factors, but also possibly T cell independent.

## B Cells: Autoantibodies in Stroke

B lymphocytes originate in the bone marrow and differentiate in the spleen. B cell activation occurs in the secondary lymphoid organs such as the spleen or lymph nodes and requires the recognition of antigen by B cell receptors (BCR) and often signaling by T helper cells. B cells progress through an antigen-independent combinatorial rearrangement of the variable (V), diversity (D) and joining (J) gene segments. This leads to the development of a vast repertoire of VDJ segments encoding the BCR, capable of recognizing a huge diversity of antigens (77). Autoreactive B cells initially constitute 75% of immature B cells and undergo several selection processes in the bone marrow and spleen to establish self-tolerance (78). Negative selection occurs in B cells that recognize self-antigen with their B cell receptor (BCR); they undergo apoptosis, receptor editing, or anergy leading to a state of central tolerance lacking pathological autoreactivity. However, these self-tolerance mechanisms fail to delete all B cells reactive to brain antigens. Brain-reactive autoantibodies can be detected in 92% of human sera (79). B cells can also acquire self-reactivity through somatic hypermutation (SMH). This process follows antigen-dependent activation and allows through affinity maturation, the diversification of B cell receptors through mutations within the variable gene regions of immunoglobulins (80). B cells can also change their constant region of their immunoglobulin heavy (IgH) chain referred to as IgH class switch recombination (CSR). Naive mature B cells produce both IgM and IgD, which are the first two heavy chain segment in the immunoglobulin locus. After activation and T cell dependent cytokine signaling, they undergo antibody class switching to produce IgG, IgA, or IgE antibodies (80). IgM antibodies are the first antibodies to respond to antigen exposure and are associated with primary immune response. IgG are associated with the secondary immune response and represent the vast majority of serum antibodies found in humans. They are major components of humoral immunity involved in controlling infections and neutralizing pathogens and toxins.

The production of autoantibodies requires the antigen-dependent activation of B cells following ischemia. Brain antigens need to bind to the BCR of an autoreactive B cell. They are then taken up in to the B cells by receptor-mediated endocytosis, degraded, and presented by MHC-II molecules on the cell membrane (81). In the case of T cell dependent activation, autoreactive T cells activated by the same antigen, would need to bind to MHC-II through their T cell receptor (TCR) followed by expression of co-stimulatory factors and cytokines which promote B cell proliferation, somatic hypermutation and class switching. The expression of costimulatory molecules can be increased following infection (60) and the release of damage-associated-molecular patterns (DAMPs) after stroke (82). In the case of T cell independent activation, B cells are stimulated by antigens such as pathogen associated molecular patterns (PAMPs), like bacterial polysaccharides or extensive cross-linking of BCRs to repeated epitopes of an antigen. Pathogenic antigens can be increased because of post-stroke infection which is a common occurrence due to stroke-induced immunosuppression (83, 84). The pathway of brain antigen release, as described in the T cell section above, would allow antigen-dependent activation to take place in the CNS and the secondary lymphoid tissue such as the cervical lymph nodes (5).

There is evidence of an immunoglobulin production in the cerebrospinal fluid (CSF) of patients with cerebrovascular disease (85, 86). In the CSF of 318 stroke patients IgG, IgM, and IgA immunoglobulin synthesis was significantly elevated in 24.8% of patients and 17.9% had CSF-specific oligoclonal IgG band, compared to 2.5% in age- and sex-matched controls (87). The absence of oligoclonal bands in the serum of stroke patients suggests an additional autoimmune B cell activation in the CNS outside of the cervical lymph nodes in the weeks after stroke (83). Activated B lymphocytes producing IgG, IgM, and IgA could be found in the ischemic mouse brain adjacent to the lesion beginning 2 weeks following stroke with extensive infiltration at 7 weeks. The B cells were surrounded by cells expressing the dendritic cell marker CD11c, which suggests antigen-presentation. B cells that were clustered with T lymphocytes were compartmentalized, suggestive of structures similar to ectopic B cell follicle with germinal centers (40, 83). In autopsy brain tissue of 21 stroke patients, more B cells, and IgG positive antibodies were found than in controls (40). In other studies, elevated autoantibodies to brain specific neurofilaments (NF) were found in the serum of stroke patients in the first 6 months, whereas antibodies to the ubiquitous antigen, cardiolipin (CL) were not elevated (88).

There is also evidence of autoantibodies following stroke that bind to the N-methyl-D-aspartate receptor (NMDAR). The condition of Anti-NMDA-receptor encephalitis is mediated by autoantibodies that target the GluN1 (NR1) subunit of NMDA receptors in brain (89). The serum levels of autoantibodies to the GluN2 (NR2) subtype of NMDA receptors were elevated within 3 h and peaked at 10–12 h in patients after stroke or TIA (90). Patients with elevated GluN2 (NR2) antibodies before cardiac surgery were 18 times more likely to experience a postoperative neurological event such as confusion, TIA or stroke than following a negative NR2 antibody test (91). This could be due to an already compromised cerebrovascular circulation and ongoing silent ischemia. Accordingly, the presence of serum autoantibodies to the NMDA GluN2 (NR2) receptor showed a correlation with risk factors for stroke such as diabetes, hypertension or recent stroke/TIA (92). In another study, antibodies against the GluN1-S2 subunit of the NMDA receptor were found in the serum of 44% of 48 stroke patients compared to 3% in age matched healthy controls within 48 h of stroke onset. The presence of these antibodies was associated with worse clinical state on presentation, larger infarcts and cortical involvement (93). In contrast, increased immunoreactivity to the NMDA GluN2 (NR2) subunit in lymphoid tissue was associated with smaller infarcts within the first week of stroke (5). The early detection of increased autoantibodies levels within hours after stroke onset is suggestive of a rapid amplification of naturally occurring autoantibodies following stroke related antigen exposure (94). The role of naturally occurring IgM antibodies to annexin IV and a subset of phospholipids, was assessed by Elvington et al. (95). Ischemia reperfusion injury was mediated through the postischemic binding of natural IgMs to neoepitopes in the infarct area with complement activation and a proinflammatory phenotype in a T cell independent process.

## B Cells–Beneficial or Detrimental?

In order to assess the beneficial or detrimental effect of B cells, the time after stroke and the different B cells subsets need to be considered. Regulatory B cells constitute only 0.5–0.7% of CD19+ B cells (96) and have been associated with protective effects following stroke. B cell deficient mice that received B cells from IL 10^−/−^ mice had larger infarct volumes, more severe functional deficits, higher mortality and increased numbers of activated T cells, macrophages, microglial cells, and neutrophils in the affected brain 48 h after stroke induction in the MCAO model (97). B cell deficient mice that received IL-10+ B cells had reduced infarcts, fewer infiltrating cells and a significant increase in regulatory T cells (98). The intrastriatal injection of cells, containing all CD19^+^ B cell subsets, into B cell deficient mice reduced infarct volumes 48 h after stroke onset. This would suggest a strong role for the default mixture of B cells to favor immunoregulation over inflammation early after experimental stroke (96). Conversely, (8) found B cells to play only a minor role in acute ischemia and (40) found no difference in infarct volumes at 72 h in B cell deficient mice. In the same study, a delayed detrimental role of B cells was discovered, with cognitive impairment within 7 weeks of stroke as a possible cause of post-stroke dementia. Delayed cognitive impairment was circumvented in B cell deficient mice and wild-type mice by B cell depletion with an antibody 5 days after stroke (40). In a cohort of 58 stroke patients, elevated titers of serum antibodies to MBP were associated with cognitive decline in the year after stroke (99). In other models, an early protective effect of regulatory B cells and a delayed detrimental effect of B cells have been found. In EAE, regulatory B cells have only shown a protective anti-inflammatory role during early EAE induction. On the other hand, B cell depletion during disease expansion reduced the infiltration of encephalitogenic T cells within the CNS and draining lymph nodes and inhibited MOG-specific T cell expansion, dramatically reducing disease symptoms (100). In experimental brain injury models, such as spinal cord lesions, B cell deficient mice have shown improved locomotor function, and reduced lesion pathology compared with wild-type mice that had antibody producing B cells and IgGs in their CSF or injured spinal cord (101, 102).

Taken together there is evidence for naturally occurring brain-reactive antibodies that escape tolerance mechanisms prior to stroke onset. Antibody titers and their presence are dramatically increased in the first hours and days following stroke. This would suggest a rapid augmentation of naturally occurring autoantibodies upon encountering increased levels of brain antigen. The delayed detection of autoantibodies and clusters of B cells and T cells in brain lesions could indicate a stroke-related generation of novel autoantibodies. There are conflicting results regarding the detrimental or beneficial effects of brain-reactive autoantibodies in the acute phase of stroke and further studies are needed. Finally, regulatory B cells seem to be protective early in stroke, while delayed detrimental effects have been shown for B cells.

## Brain Trauma

Traumatic brain injury (TBI) involves a primary injury with multifocal damage to axons, glia, dendrites, and a microvasculopathy with microbleeds, endothelial damage, and breach of the blood brain barrier (BBB) (103, 104). Similar to stroke, it is followed by a secondary injury involving an inflammatory reaction with pro-and anti-inflammatory cytokines, microglial activation, invasion of peripheral neutrophils, lymphocytes and macrophages (105), damaging levels of glutamate (106), and free radicals (107, 108).

Biomarkers that have been identified in human and animal biofluids after TBI include, glial fibrillary acidic protein (GFAP), myelin basic protein (MBP), neuron specific enolase (NSE), glia calcium-binding protein S100B, ubiquitin carboxyl hydrolase-like 1 (UCHL1), αII-spectrin fragments, and neurofilament proteins (109–115). The release of these brain proteins from injured cells into the bloodstream and the aforementioned neuroinflammatory response can trigger an autoimmune response with the generation of autoantibodies.

## Autoantibodies in TBI

The presence of long-term hypopituitarism after TBI, most frequently growth hormone (GH) deficiency, has led researchers to investigate anti-hypothalamus antibodies (AHA), and anti-pituitary antibodies (APA), showing significant associations between these antibodies and head trauma (116, 117). High titers of APA and AHA in boxers were associated with hypopituitarism even after 5 years following TBI (118). The pathophysiological pathway of hypopituitarism and the role of autoimmunity in changes of hormone levels remains to be investigated.

S100B is a calcium-binding protein, present in perivascular astrocytes, but also in the CNS as an astrocyte-specific protein. S100B increases in the CSF and serum in correlation to the severity of traumatic brain injuries within the first hours to days (115, 119, 120). In football players experiencing repetitive sub-concussive head hits, increased levels of S100B could be detected following individual games. Players with the most significant elevations of S100B had the highest titer of serum S100B autoantibodies at the end of a season. The level of S100B autoantibodies predicted MRI abnormalities and correlated with cognitive changes (121). S100B leakage into serum, could therefore have contributed to the development of autoantibodies after one season. MBP autoantibodies have been measured in the CSF of patients with a strong correlation to the severity of injury, which was measured by the Glasgow Coma Scale (GCS) (122). GFAP is an intermediate filament protein, predominantly found in the cytoskeleton of astrocytes. GFAP autoantibody levels increased by 7 days after TBI in humans (123) and were also found at a chronic time point 6 months post-injury (124). There were also higher plasma autoantibody levels against GFAP in patients with acute TBI and previous exposure to TBI compared to those without a previous history of TBI (124). Serum levels of autoantibodies against AMPA glutamate receptors (GluR1 subunit) and NMDA glutamate receptor (NR2A subunit) were examined in the serum from 60 children (7–16 years) with chronic posttraumatic headache at 6 and 12 months following trauma. Antibody titers to AMPA and largely NMDA were found to be higher when neurological symptoms persisted in children, which according to the authors was suggestive of hypoxic brain lesions, and evidence of hyperstimulation of NMDA glutamate receptors with greater permeability of the blood-brain-barrier (125).

Taken together, elevated levels of brain-reactive autoantibodies can also be found following clinical TBI, in many cases correlating to the severity of injury and frequency of head impacts. The presence of autoantibodies does not provide evidence for an autoimmune disease. The pathogenic potential of brain-reactive antibodies, that could enter the brain through a compromised BBB remains to be explored.

## Adaptive Immunity in TBI

The specific role of neuroinflammation and autoantibodies in the recovery and neuropathology of TBI patients remains unclear. TBI induced neuropathology with accumulation of proteins such as amyloid beta (Aβ) has been associated with an increased risk of neurological diseases such as Alzheimer's disease (AD) and chronic traumatic encephalopathy (CTE) (126). The question whether chronic neuroinflammation causes this neuropathology or if it is a response to abnormal protein accumulation is not understood (105). In a recent study, concussion in adolescence was associated with an increased risk of multiple sclerosis (127). Experimental evidence points to a link between injury to the nervous system and myelin-antigen-specific T cells, that have been shown to result in encephalomyelitis, if the genetic predisposition of an autoimmune disease is present (128). In one study reactive microglia were found in 28% of patients with traumatic brain injury with survival of >1 years and were linked to white matter degeneration and thinning of the corpus callosum (129). In another study, microglial activation was associated with more severe cognitive impairment years after trauma (130). Early T cell infiltration, peaking as early as 3–24 h after experimental TBI has been described in previous studies (131, 132). In another experimental study intracerebral injection of ovalbumin led to the infiltration and accumulation of ovalbumin-specific CD8+ T cells at sites of the cognate antigen. Following TBI, these activated antigen-specific CD8+ T cells were also attracted to sites of traumatic injury. This process could not be reduced by blocking CD154, which is presumed to be important for antigen-specific T cell activation, pointing to a presumably antigen-independent attraction to injury (133). In an experimental study of weight-drop induced closed head injury, Rag1^−^/^−^ mice deficient of B and T lymphocytes, showed no difference regarding neurological and histological outcome and inflammatory mediators for up to 7 days following injury, compared with wild-type mice (134). In a similar study of weight-drop TBI, FTY720 was used to significantly reduce the numbers of circulating and brain infiltrating lymphocytes. There was no difference between BBB integrity, lesion size or functional outcome at 24 up to 7 days compared to untreated mice (135). This suggests that early T cell recruitment, which would presumably be antigen-independent, plays no role in early TBI up to 1 week. MBP autoreactive T cells were found in 40% of 10 patients 10 days after TBI using proliferation assays, but with no correlation to injury volume or severity in one study (136). The role of lymphocytes in TBI mediated inflammation and recovery, the question of antigen-specificity and activation pathways, especially in chronic models of TBI remain to be investigated.

## Adaptive Immunity in Other CNS Lesion Models

In others models of injury to the CNS, there is evidence for a protective role of specific T cells. In optic nerve injury passively transferred T cells have been shown to accumulate at the injury site between 3 and 21 days irrespective of their antigen-specificity (MBP vs. ovalbumin) (137). The transfer of T cells specific to MBP to mice with optic nerve injury however promoted recovery, while this effect could not be observed with activated T cells to other non CNS-antigens (e.g., ovalbumin) (138). Mice that were actively immunized with myelin associated peptides such as MOG before injury to the optic nerve also had less degeneration of retinal ganglion cells (139). Similarly inducing tolerance to myelin proteins in neonates decreased the ability to a protective T cell mediated immune response to injury of myelinated axons in the CNS (140). Autoreactive T cells to myelin proteins such as MBP have also been found significantly proliferated in clinical and experimental spinal cord injury (SCI) (141–144) together with increased levels of serum autoantibodies against MAG and GM1 ganglioside in patients (145). In a model of facial nerve injury an expansion of myelin-antigen-specific T cells secreting IFN-y in cervical lymph nodes could be seen after 8 days (146). The transfer of anti-MBP T cells or the active immunization with MBP at the time and 1 week after contusion in an experimental rat model of SCI in both cases resulted in a significantly better functional outcome (147). In contrast, endogenous MBP-reactive lymphocytes, activated by experimental traumatic SCI contributed to tissue injury and impaired functional recovery (148). In a model of MBP (63–68) peptide induced EAE, encephalitogenic T cells using the TCR-BV8S2^+^ had a much higher proinflammatory cytokine to neurotrophin ratio than lymphoid cells bearing other TCRs. This would suggest that neuroprotection could be mediated by other non-MPB peptide-specific lymphoid cells (149, 150).

The question of the neuroprotective role of T cells and their antigen-specific activation was further addressed in a study with optic nerve crush injury and spinal cord injury. CD4+ T cells were activated independently of MHC II via damage-associated molecular mediators from CNS tissue that induced a population of neuroprotective IL-4 producing T cells. Recovery was promoted via neuronal IL-4 receptors that potentiated neurotrophin signaling (151). This suggests that antigen-independent pathways also play a role in neuroprotection after CNS injury. The question whether this protective mechanism is later enhanced by an autoimmune T cell response or if T cell infiltration could be increased through vaccination with an appropriate CNS self-antigen remains to be explored (139).

Recently, it was shown, that autoantibodies directed against different nervous system and systemic self-antigens, that increased within 5 days of human SCI, were already present in healthy subjects (152). Similar to stroke, this is an indication of a rapid augmentation of pre-existing naturally occurring autoantibodies. The role of autoreactive B cells has been investigated in SCI-induced autoimmunity (101, 102, 153). SCI induces B cell proliferation, activation, and differentiation into IgG+ plasma cells. The formation of large B cell clusters expressing MHC II surrounded by CD3+ T cells and increased IgG synthesis after SCI resembles lymphoid follicles with B cell and T cell interactions (101). There is a delayed accumulation of B cells in the CSF and injured spinal cord with large deposits of antibody at sites of axon pathology and demyelination (102). Injection of circulating IgG antibodies purified from SCI mice in the spinal cord of uninjured animals causes consistent paralysis and pathology and B cell deficient mice have smaller lesions and better functional outcome after SCI (102).

## Conclusion

Stroke and Traumatic Brain Injury (TBI) cause the release of brain-derived antigens into the circulation. In stroke, the detection of autoreactive T cells and the presence of autoantibodies to brain antigens suggests the possibility of autoimmunity. Inducing immune tolerance to brain antigens prior to experimental stroke shows mostly beneficial effects. Increased immunoreactivity to brain antigens and brain-reactive antibodies is associated with mostly detrimental effects, but some beneficial effects have also been shown. The early immunoreactivity to brain-antigens and detection of elevated levels of autoantibodies within hours to days after stroke onset, suggests the expansion of naturally occurring autoantibodies and pre-existing self-reactive T cells. The delayed presence of B cell and T cell clusters in stroke lesions with antibody production correlating to cognitive decline could be an indication of a detrimental adaptive immune response. The proinflammatory microenvironment and infections following stroke could favor autoimmune responses to brain antigens breaking tolerance mechanisms. In TBI, the levels of brain antigens and brain-reactive antibodies correlate with the severity of stroke and frequency of head impacts in most studies. If these antibodies are pathogenic, remains to be further explored. There is little evidence for the early role of T cells in early TBI and their long-term effects need to be investigated. In optic nerve crush injury and spinal cord lesion models, autoreactive T cells show protective effects, while antibody producing B cells exacerbate spinal cord injury. Taken together, the existing data in favor of autoimmunity remains inconclusive. The effect of self-reactive autoantibodies is possibly small compared to other detrimental effects of the lesion itself and the induced local inflammation. Further studies are needed to understand the role of autoimmunity after brain lesions, investigating the generation, function and pathway of autoantibodies and the extent of their protective or detrimental role, with possible therapeutic implications.

## Author Contributions

EJ prepared and wrote the manuscript. TM wrote and supervised the manuscript.

### Conflict of Interest Statement

The authors declare that the research was conducted in the absence of any commercial or financial relationships that could be construed as a potential conflict of interest.
